# Current status of head and neck sarcomas in Japan in 2016–2019: an analysis using the national cancer registry

**DOI:** 10.1007/s10147-024-02484-5

**Published:** 2024-02-27

**Authors:** Ryoko Rikitake, Yu Mizushima, Seiichi Yoshimoto, Takahiro Higashi, Tomoyuki Satake, Chigusa Morizane, Akira Kawai

**Affiliations:** 1 Department of Public Health and Health Policy, Graduate School of Medicine, The University of Tokyo, 7-3-1 Hongo, Bunkyo-Ku, Tokyo, 113-0033 Japan; 2 Institute for Cancer Control, Division of Health Services Research, National Cancer Center, Tokyo, Japan; 3 Rare Cancer Center, National Cancer Center, Tokyo, Japan; 4 Department of Head and Neck Oncology, National Cancer Center Hospital, Tokyo, Japan; 5 Department of Hepatobiliary and Pancreatic Oncology, National Cancer Center Hospital, Tokyo, Japan; 6 Department of Musculoskeletal Oncology and Rehabilitation Medicine, National Cancer Center Hospital, Tokyo, Japan

**Keywords:** Epidemiology, Head and neck cancer, Sarcoma, Rare cancer, National Cancer Registry

## Abstract

**Background:**

Head and neck sarcomas are especially rare in Asia, leading to limited clinical evidence. This study aimed to investigate the incidence, clinical features, treatment status, and outcome of these sarcomas using data from the National Cancer Registry in Japan.

**Methods:**

All head and neck sarcomas diagnosed between 2016 and 2019 and recorded in the National Cancer Registry were analyzed. Data on sex, age, primary site, histological type, stage, treatment modality, and prognostic information were collected. Age-adjusted incidence and 3-year survival rates of patients with head and neck sarcomas were calculated.

**Results:**

Overall, 635 head and neck sarcoma patients were identified. Head and neck sarcoma occurred more frequently in men and patients in their 70 s. The age-adjusted annual incidence rate was 0.125 per 100,000 patients in the 2015 Japanese model or 0.089 per 100,000 patients in the world population model. The nasal cavity and paranasal sinuses were the most frequent primary sites, with rhabdomyosarcoma as the most common histologic type. Treatment typically involved chemotherapy and/or radiation therapy for rhabdomyosarcoma and Ewing’s sarcoma, whereas surgical approaches for other types. Three-year survival rate of head and neck sarcoma patients was 64.8%.

**Conclusions:**

Head and neck sarcomas occurred rarely, but most frequently in the nasal cavity and paranasal sinuses in Japan. Poor outcomes were observed for sarcoma patients than for non-sarcoma head and neck cancer patients.

## Introduction

Cancers that arise from the head and neck (H&N) regions form a heterogeneous group due to the diversity of functional organs in this anatomical area. According to global statistics (GLOBOCAN 2020), H&N cancers account for approximately 5% of all cancers and are the 7th most common cancers, with the most common site of oral cavity (including lip) [1]. Geographical and epidemiological differences in the incidence and clinical characteristics of H&N cancers have been reported [2–4]. For example, more than 70% of new cases of nasopharyngeal cancer occurred in East and Southeast Asia [5].

Sarcomas comprise approximately 1% of all H&N cancer cases in the United States [6]. In Asia, the exact incidence and treatment status of H&N sarcomas are largely unknown. For the development of new strategies to effectively treat rare diseases, information on basic characteristics and epidemiology for each region is essential.

In 2016, Japan initiated a nationwide population-based cancer registry, the National Cancer Registry (NCR), which collects data on cancer cases reported by all hospitals in Japan, as mandated by legal requirements. This study aimed to comprehensively describe the incidence, clinical features, treatment modalities, and survival outcomes of H&N sarcomas using the NCR database in Japan.

## Patients and methods

### Data source and study population

We used data from the NCR to calculate the annual incidence and treatment modalities of H&N sarcomas. Since the launch of the NCR in 2016, it included cases diagnosed between 2016 and 2019. As the 5-year survival data were not available at the time of analysis, we analyzed 3-year survival data instead. Patients with malignant lymphoma and Kaposi’s sarcoma were excluded. We extracted cancer cases of H&N regions (lip and oral cavity, nasopharynx, oropharynx, hypopharynx, larynx, major salivary gland, nasal cavity and paranasal sinuses, and middle ear) using the International Classification of Diseases for Oncology (third edition) topography (C00x-C14x, C30x-C32x, and C442) and histology (8010–9582) codes. The following histology codes of H&N sarcomas were used: 8710, 8711, 8800–8814, 8816–8829, 8831–8939, 8942–8981, 8983–9044, 9120–9132, 9134–9139, 9141–9149, 9151–9262, 9364, and 9540–9582. We focused on H&N regions typically treated by Japanese H&N surgeons [7], excluding the scalp or skin regions from our analysis.

The following information was extracted from the NCR database: age, sex, initial treatment method (surgery, radiation therapy [RT], chemotherapy, and/or others), detection route (screening, incidental, autopsy, or others), summary stage (carcinoma in situ, localized, regional lymph node metastasis, invasion to adjacent structures, distant metastasis, or unknown), and prognostic information. Standard population data for the Japanese population in 2015 were obtained from the Ministry of Health, Labour, and Welfare, Japan.

### Data analyses

The incidence of respective H&N cancer types in the population was calculated as the number of new cases of H&N cancers divided by the total number of individuals in the Japanese population in 2016. Both crude and age-adjusted incidence rates were reported. Age-adjusted incidence rates were determined using weighted proportions of the corresponding age groups according to the 2015 Japanese standard population or the World Health Organization (WHO; 2000–2025) world standard population. The 3-year survival rates with 95% confidence intervals (95% CIs) of patients with H&N sarcomas and those without sarcomas treated between 2016 and 2017 were calculated. Overall survival was estimated using Kaplan–Meier methods. Proportions of the examined factors across groups were statistically analyzed using χ2 tests. The differences in survival curves were statistically examined using the log‐rank test. All statistical analyses were performed using Stata MP16.1 (College Station, TX, USA). A p-value of less than 0.05 was considered statistically significant.

### Ethical considerations

According to the procedure stipulated by the Cancer Registry Act, the protocol for the NCR analysis was reviewed by the Data Utilization Committee of the National Cancer Registry Office. As per the research ethics guidelines in Japan, our study was exempt from a review by our institutional review board because data provision and handling complied with legal procedures. While the data were extracted from the NCR database, the authors have sole responsibility for the analysis and interpretation of the findings.

According to the privacy protection rule of cancer registries, the numbers of cases less than 10 (except for 0) were presented as “ < 10” for the NCR data.

## Results

### Incidence and treatment of H&N sarcomas

A total of 635 patients with H&N sarcomas diagnosed between 2016 and 2019 were identified from the NCR database. The number of patients without sarcomas registered in the NCR in the same period was 126,867, of which 104,095 patients had H&N squamous cell carcinoma. H&N sarcomas occurred more frequently in men (58.4%) than in women (41.6%). The most frequent age category of patients was the 70 s (Table 1). The nasal cavity and paranasal sinuses were the most frequent primary sites (n = 252, 39.7%), followed by the oral cavity (including the lip; n = 220, 34.7%). The crude incidence rate for H&N sarcomas and H&N cancer as a whole was 0.13 and 25.01 per 100,000 patients, respectively. The age-adjusted incidence rate for H&N sarcomas was 0.125 per 100,000 patients in the 2015 Japanese model or 0.089 per 100,000 patients in the WHO (2000–2025) world population model. Table 2 shows the different histological types. The most common histological types were rhabdomyosarcomas (n = 134, 21.1%), osteosarcomas (n = 64, 10.1%), and chondrosarcomas (n = 54, 8.5%). Table 3 presents the tumor types according to the primary site. Rhabdomyosarcoma (92 cases), leiomyosarcoma (18 cases), and Ewing’s sarcoma (16 cases) occurred more commonly in the nasal cavity and paranasal sinuses than in other primary locations. Meanwhile, osteosarcoma and chondrosarcoma were more common in the oral cavity (53 cases) and larynx (26 cases). Table 4 summarizes the treatment modalities according to histological type. Rhabdomyosarcoma (71 cases) and Ewing’s sarcoma (12 cases) were mainly treated with chemotherapy and/or RT, whereas the other subtypes tended to be mainly treated with surgery.

**Table 1:** Characteristics of patients with head and neck sarcomas

Characteristic	n (%)
Sex	
Male	371 (58.4)
Female	264 (41.6)
Age	
0–9	18 (2.8)
10–14	18 (2.8)
15–19	10 (1.6)
20–24	26 (4.1)
25–29	20 (3.2)
30–34	28 (4.4)
35–39	18 (2.8)
40–44	32 (5)
45–49	31 (4.9)
50–54	28 (4.4)
55–59	39 (6.1)
60–64	40 (6.3)
65–69	72 (11.3)
70–74	71 (11.2)
75–79	68 (10.7)
80–84	55 (8.7)
85–	61 (9.6)
Unknown	0 (0)
Route of detection	
Screening	< 10 (−)
Incidental	125 (19.7)
Autopsy	< 10 (−)
Other	456 (71.8)
Unknown	48 (7.6)
Summary Stage	
Carcinoma in situ	0 (0)
Localized	200 (31.5)
Regional lymph node metastasis	21 (3.3)
Invasion of adjacent structures	197 (31)
Distant metastasis	57 (9)
Unknown	160 (25.2)
Sites	
Lip and oral cavity	220 (34.7)
Nasopharynx	< 10 (−)
Oropharynx	22 (3.5)
Hypopharynx	36 (5.7)
Larynx	67 (10.6)
Major salivary gland	26 (4.1)
Nasal cavity and paranasal sinuses	252 (39.7)
Middle ear	< 10 (−)
Total	635 (100)

^*^Less than 10 cases (except for 0) of given age ranges, route of detection, and tumor sites are represented as “ < 10.”

**Table 2:** Characteristics of the histological types recorded in the National Cancer Registry between 2016 and 2019

Pathology	n (%)
Rhabdomyosarcoma	134 (21.1)
Osteosarcoma	64 (10.1)
Chondrosarcoma	54 (8.5)
Liposarcoma	44 (6.9)
Angiosarcoma	39 (6.1)
Leiomyosarcoma	33 (5.2)
Undifferentiated pleomorphic sarcoma	32 (5)
Ewing's sarcoma	28 (4.4)
Malignant peripheral nerve sheath tumor	20 (3.2)
Giant cell sarcoma**	17 (2.7)
Undifferentiated sarcoma	17 (2.7)
Fibrosarcoma	16 (2.5)
Synovial sarcoma	12 (1.9)
Myxofibrosarcoma	< 10 (−)
Epithelioid sarcoma	< 10 (−)
Others	109 (17.2)
Total	635 (100)

^*^Less than 10 cases (except for 0) of given histological types are represented as “ < 10.”

^**^ Except for M-9250/3

**Table 3:** The most and second-most frequent primary sites for each histological type recorded in the National Cancer Registry between 2016 and 2019

Pathology	Most frequent primary sites	n	(%)	Second-most frequent primary sites	n	(%)	Total
Rhabdomyosarcoma	Nasal cavity and paranasal sinuses	92	68.7	Lip and oral cavity	17	12.7	134
Osteosarcoma	Lip and oral cavity	53	82.8	Nasal cavity and paranasal sinuses	10	15.6	64
Chondrosarcoma	Larynx	26	48.1	Lip and oral cavity	16	29.6	54
Liposarcoma	Hypopharynx	18	40.9	Lip and oral cavity	17	38.6	44
Angiosarcoma	Nasal cavity and paranasal sinuses	16	41.0	Lip and oral cavity	14	35.9	39
Leiomyosarcoma	Nasal cavity and paranasal sinuses	18	54.5	Lip and oral cavity	< 10	–	33
Undifferentiated pleomorphic sarcoma	Lip and oral cavity	11	34.4	Nasal cavity and paranasal sinuses	10	31.3	32
Ewing's sarcoma	Nasal cavity and paranasal sinuses	16	57.1	Lip and oral cavity	< 10	–	28
Others	Nasal cavity and paranasal sinuses	80	41.9	Lip and oral cavity	78	37.7	207

^*^Less than 10 cases (except for 0) of given treatment modalities for each histological type are represented as “ < 10.”

**Table 4:** Treatment modalities for each histological type recorded in the National Cancer Registry between 2016 and 2019

Pathology	Operation	Operation + (Chemotherapy and/or RT)	Chemotherapy and/or RT	Others/Unknown	Total
Rhabdomyosarcoma	18	18	71	27	134
Osteosarcoma	18	20	< 10	#	64
Chondrosarcoma	36	< 10	< 10	11	54
Liposarcoma	30	< 10	0	12	44
Angiosarcoma	17	< 10	< 10	11	39
Leiomyosarcoma	15	< 10	< 10	< 10	33
Undifferentiated pleomorphic sarcoma	13	< 10	< 10	< 10	32
Ewing's sarcoma	< 10	< 10	12	< 10	28
Others	##	21	32	90	207
Total	212	95	137	191	635

*RT* radiation therapy

^*^Less than 10 cases (except for 0) of given treatment modalities for each histological type are represented as “ < 10.”

^#^For the avoidance of the calculation of “Chemotherapy and/or RT” number for osteosarcoma cases, this value is not shown

^##^For the avoidance of the calculation of “operation” number for Ewing's sarcoma cases, this value is not shown

### Survival rates of patients with H&N sarcomas

In total, 222 cases of H&N sarcoma were treated between 2016 and 2017. The 3-year survival rate of the 48,900 patients without sarcomas treated in the same period was 73.2% (95% CI 72.7–73.6%), while that of 43,182 patients with H&N squamous cell carcinoma was 72.9% (95% CI 72.5–73.4%).

**Fig. 1:**
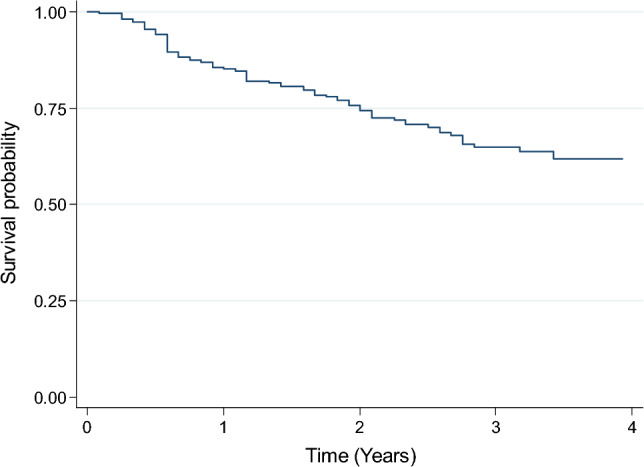
Kaplan–Meier survival curve for the total numbers of treated patients (222 cases) with head and neck sarcomas registered in the National Cancer Registry from 2016 to 2017

**Fig. 2:**
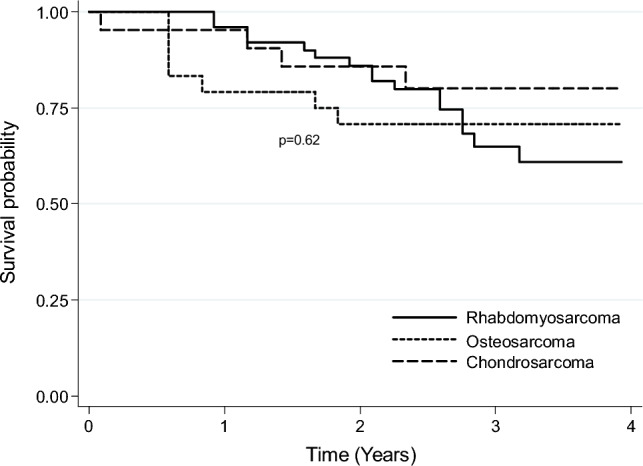
Kaplan–Meier survival curves for treated head and neck sarcoma cases with histology of rhabdomyosarcoma (50 cases), osteosarcoma (24 cases), and chondrosarcoma (21 cases) registered in the National Cancer Registry from 2016 to 2017

Figure 1 shows the Kaplan–Meier survival curve for H&N sarcoma cases treated between 2016 and 2017. The 3-year survival rate for all H&N sarcoma cases (treated cases) was 64.8% (95% CI 57.5–71.2%). Figure 2 shows the Kaplan–Meier survival curves for H&N sarcoma cases with histology of rhabdomyosarcoma (50 cases), osteosarcoma (24 cases), and chondrosarcoma (21 cases) treated between 2016 and 2017. The 3-year survival rate for H&N sarcoma cases with these histologies were 64.9% (95% CI 47.9–77.7%), 70.8% (95% CI 48.4–84.9%), and 80.0% (95%CI 54.9–92.0%), respectively.

## Discussion

In this study, we elucidated the comprehensive nationwide status of H&N sarcomas in Japan using the NCR database. National databases are useful for studying extremely rare diseases such as H&N sarcomas. In the United States, the National Cancer Institute’s Surveillance, Epidemiology, and End Results (SEER) registry and the National Cancer Database have been extensively used to analyze H&N sarcomas according to their histologic types [8]. In Europe, the Surveillance of Rare Cancers project (RARECARE) was launched to estimate the incidence of many rare cancers, including that of H&N sarcoma [9]. Moreover, In the United Kingdom, the National Cancer Intelligence Network (NCIN) has conducted research on H&N sarcomas [10], and in France, the BCB SARCOMES database was established as a national database dedicated to sarcomas [11]. We believe that the accumulation of database analyses will create a global landscape of rare and heterogeneous cancers.

In this study based in Japan, H&N sarcomas occurred more frequently in males (58.4%), consistent with the findings from other countries [8, 10]. One study reported female predominance of hard tissue sarcomas [12]. The majority of the patients were aged approximately 70 years, which was slightly higher than that reported previously (median age of approximately 50 years) [13, 14]. These variations among studies may partly reflect the geographic variation in the incidence of H&N sarcomas or may be due to the relatively small sample size of previous studies.

In the present study, the age-adjusted incidence rate of H&N sarcomas was 0.089 per 100,000 individuals in the WHO world population model. In the RARECARE project, the annual incidence of soft tissue sarcoma was reported to be approximately 5 per 100,000, while that of H&N sarcomas was 5% [15]. Therefore, the annual incidence of soft tissue sarcoma within the H&N region is approximately 0.25 per 100,000 in Europe, and a slightly smaller value was obtained in the current study in Japan. In our study, we focused on H&N regions, which are usually treated by Japanese H&N surgeons, and excluded the scalp or skin regions. Therefore, our results regarding the incidence of H&N sarcomas cannot be directly compared with those in overseas databases.

The nasal cavity and paranasal sinuses and oral cavity were the most frequent H&N primary sites in the current study. One study of a sarcoma registry including 214 H&N sarcoma cases in the United States showed that the major sites were the parotid and neck (20%), face and forehead (18%), maxilla and palate (13%), scalp (12%), mandible (11%), paranasal sinuses (7%), larynx (2%), and oral cavity (5%) [16]. However, owing to previously mentioned reasons, our results regarding the primary locations of H&N sarcomas cannot be directly compared with those of overseas studies.

The most common histologic types in the Japanese population were rhabdomyosarcoma, osteosarcoma, and chondrosarcoma. A SEER-based analysis reported that the most common histological types in adult patients were malignant fibrous histiocytoma/undifferentiated pleomorphic sarcoma (MFH/UPS), Kaposi’s sarcoma, and hemangiosarcoma, while rhabdomyosarcoma, MFH/UPS, and osteosarcoma were the most common types for pediatric patients [8]. Additionally, according to the NCIN database of the United Kingdom, leiomyosarcoma, angiosarcoma, and rhabdomyosarcoma were the most common tumors arising in the connective and soft tissues of the H&N region [10]. In Africa, Kaposi’s sarcoma, which is related to human immunodeficiency virus/acquired immunodeficiency syndrome, is considered the most common H&N sarcoma [17]. In Australia, MFH/UPS is the most frequent histology, with ultraviolet light being one of the etiologic factors [18]. We consider that these regional variations in the most frequent primary histology are another facet of the geographical diversity of H&N sarcomas.

Rhabdomyosarcoma, Ewing’s sarcoma, and leiomyosarcoma are commonly observed in the nasal cavity and paranasal sinuses, while osteosarcoma and chondrosarcoma are common in the oral cavity and larynx. However, data on the trends in tumor sites for each histology are scarce, because all histological types of H&N sarcoma are exceedingly rare [19, 20]. The current study presents new evidence on the association between common sites and histological types.

Concerning treatment, rhabdomyosarcoma and Ewing’s sarcoma tended to be treated with chemotherapy and/or RT, whereas the other types were mainly treated with surgery. Surgery is the most widely indicated treatment for H&N sarcomas [21, 22]. For rhabdomyosarcoma and Ewing’s sarcoma, the 2016 guidelines for the management of pediatric cancer in Japan recommend systemic therapy and RT followed by surgery owing to surgical difficulties for extensive resection [23]. Another study concluded that all patients with H&N rhabdomyosarcoma, regardless of age, required adequate RT [24]. Therefore, our results indicate that an evidence-based approach has been widely adopted for H&N sarcoma care in Japan.

In this study, the overall 3-year survival rate for patients with H&N sarcomas was 64.8% in this study. The 3-year survival time was relatively short in H&N sarcoma cases. This is partly due to the aggressive nature of the tumor. In the SEER database study, the cause-specific 2- and 5-year survival rates for adult patients with H&N sarcomas were 76% and 66%, respectively [8]. In other studies, the 5-year survival rate for patients with H&N sarcomas ranged from 30 to 80%. In addition, patient age, tumor size, and tumor grade significantly influenced the survival of patients with H&N sarcomas [25, 26]. Patients with H&N sarcomas primarily succumb to local recurrence [27].

In the current study, we calculated only 3-year survival rates for cases with H&N sarcoma. Moreover, it is important to note that sarcomas account for relatively fewer cases (n = 222) among all other types of H&N cancers (n = 48,900), especially when compared with the number of squamous cell cancer cases (n = 43,182). The number of patients with each sarcoma histology was relatively small, making it difficult to compare prognoses for each histology or treatment modality due to the wide 95% CIs. Any findings of survival in patients with H&N sarcomas in this study may be affected by factors other than malignancy, such as the presence of greater comorbidities in these patients.

This study has some limitations. First, the observation period was limited to the research period, which did not include the latest results. Second, the 5-year survival data were not available in the current study. Longer observations might provide different views on the prognosis of H&N sarcomas. Third, the NCR does not provide information on the order of treatment (surgery, chemotherapy, and RT) or the target of RT. Therefore, an in-depth analysis or comparison of advanced cases that are usually managed with multimodal treatment could not be conducted. Finally, the NCR does not provide information on the causes of death. Therefore, only overall survival was analyzed in this study. The main purpose of the NCR is to capture the entire incidence of both common and rare cancers in Japan. The NCR is especially useful for developing cancer prevention and/or cancer screening strategies. However, there are limitations in providing clinical details for each cancer entity, such as treatment or prognosis. We speculate that the hospital-based cancer registries of cancer care hospitals designated by the national government could can overcome these limitations, provide comprehensive information on nationwide clinical practice, and elucidate clinical issues for any cancer entities.

In conclusion, we provided comprehensive and detailed information on the clinical status of rare H&N sarcomas using the NCR database in Japan. Further analysis of rare cancers is warranted for a better understanding of the disease and for improvements in standardized diagnosis and treatments.
